# Tetra­aqua­[3-oxo-1,3-bis­(pyridinium-2-yl)propan-1-olato]nickel(II) tribromide dihydrate

**DOI:** 10.1107/S205698902000081X

**Published:** 2020-01-31

**Authors:** Barry L. Westcott, Guy Crundwell, Nilda L. Alicea-Velázquez

**Affiliations:** a Department of Chemistry & Biochemistry, Central Connecticut State University, 1619 Stanley Street, New Britain, CT 06053, USA

**Keywords:** crystal structure, di­pyridyl­propane­dione, pyridinium, nickel

## Abstract

The crystal structure of the title compound, [Ni(C_13_H_11_N_2_O_2_)(H_2_O)_4_]Br_3_·2H_2_O, contains an octa­hedral Ni^II^ atom coordinated to the enol form of 1,3-di­pyridyl­propane-1,3-dione (dppo) and four water mol­ecules. Both pyridyl rings on the ligand are protonated, forming pyridinium rings and creating an overall ligand charge of +1. The protonated nitro­gen-containing rings are involved in hydrogen-bonding inter­actions with neighoring bromide anions. There are many additional hydrogen-bonding inter­actions involving coordinated water mol­ecules on the Ni^II^ atom, bromide anions and hydration water mol­ecules.

## Abstract 2

The reaction of 1,3-dipyridyl-1,2-propane-1,3-dione (dppo) with nickel(II) bromide in HBr-acidified methanol produces a bromide salt containing the [(C_13_H_11_N_2_O_2_)(H_2_O)_4_Ni]^+3^ cation. The ligand is in its enol form— the O atoms bond to the nickel whereas the pyridyl N atoms are protonated as pyridinium rings. The nickel completes its octa­hedral coordinatin sphere by binding four waters. Three bromides balance the charge of the cation and there are two waters of hydration. The mean plane of the flat ligand makes an angle of 19.480 (17)° with respect to the plane defined by the nickel and its four equatorial O atoms. There is exstensive hydrogen bonding involving the anions, waters, and pyridinium N atoms.

## Chemical context   

We chose to study 1,3-di­pyridyl­propane-1,3-dione (dppo) in our ongoing investigations of bridged dipyridyl compounds as ligands for transition metals and rare earths. Previous studies of the di-2-pyridyl ketone (dpk) ligand illustrated that it can undergo a Lewis acid assisted hydration reaction at the ketone to form a diol (Sommerer & Abboud, 1993 ▸). This hydration can also occur with Arrhenius acids; however, in the absence of a metal for coordination, the pyridyl N atoms of the resulting diol are protonated (Sommerer *et al.*, 1994 ▸). For the dppo in this study, the coordination to the metal center required the presence of an Arrhenius acid (HBr). No hydration of the dione occurred, the ligand adopted the enol form where O atoms behaved as a bidentate ligand, and protonation of the pyridyl rings was observed.
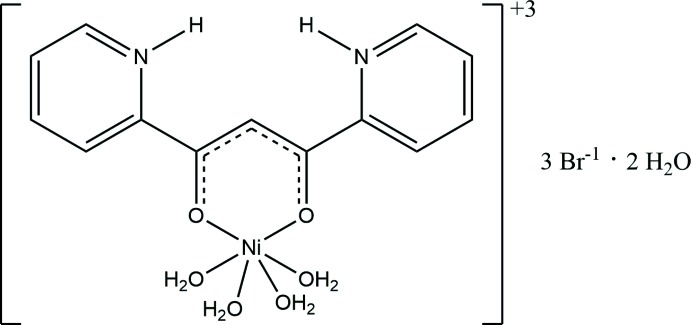


## Structural commentary   

**Figure 1:**
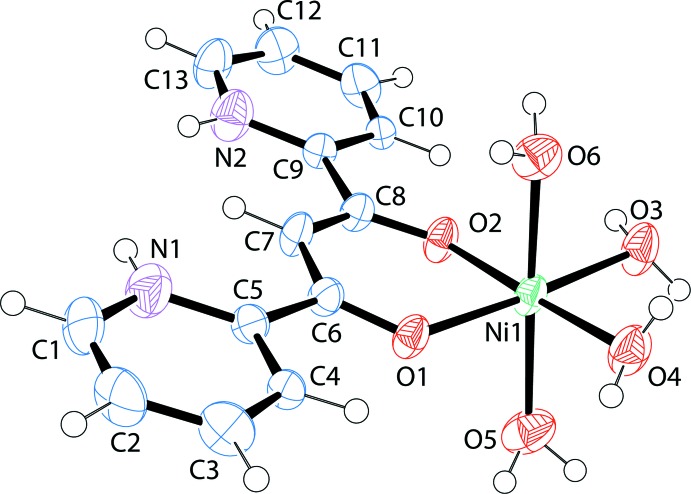
A view of the the title compound, with displacement ellipsoids drawn at the 50% probability level.

Since the synthesis of the complex was in hydro­bromic acid in methanol, the existence of three bromide anions required a trivalent cation. Planar dppo is in its enol form allowing the O atoms to behave as Lewis bases to the nickel center; however, the pyridine rings are both protonated. The H atoms were readily found in difference maps and refined as unconstrained atoms. The organic ligand therefore has an overall +1 charge. There are also four water molecules coordinated to the Ni^II^ atom, thereby completing the octa­hedral geometry of the [Ni(C_13_H_11_N_2_O_2_)(H_2_O)_4_]^+3^ cation (Fig. 1 ▸). During refinement, two additional waters of hydration were located. There is an angle of 19.48 (7)° between the mean plane of the dipyridinium ligand and the plane defined by the Ni^II^ atom and its four equatorial O atoms. Selected geometric parameters are listed in Table 1 ▸.

## Supra­molecular features   

**Figure 2:**
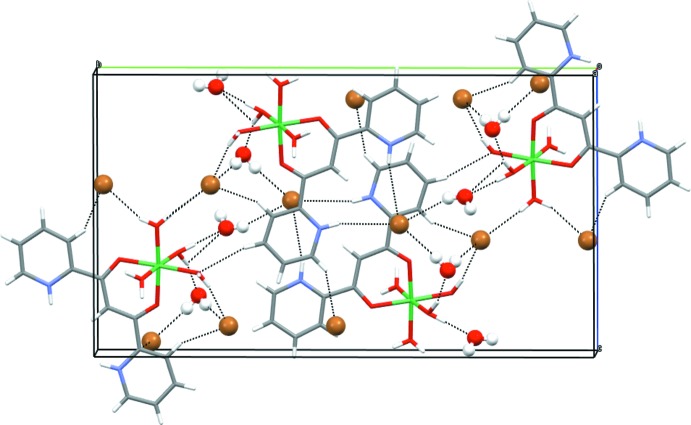
A view of the unit cell along (100). Bromines and free water mol­ecules are shown as balls and sticks and hydrogen bonds as black dashed lines (Macrae *et al.*, 2020 ▸).

A packing diagram of the compound as viewed down (100) is shown in Fig. 2 ▸. There are many hydrogen-bonding inter­actions. The pyridinium H atoms are involved in hydrogen bonding with one of the bromide anions. Bromide anions are also engaged in hydrogen bonding with the waters of hydration and the water molecules coordinated to the Ni^II^ atom. The waters of hydration extend the hydrogen-bonding network by also inter­acting with the water molecules coordin­ated to the Ni^II^ center. A summary of the hydrogen-bonding inter­actions is listed in Table 2 ▸.

## Database survey   

The enol form of dppo has been used to make extended structures with cadmium (Tan *et al.*, 2012 ▸), as well as with manganese (Langley *et al.*, 2010 ▸). The cadmium structure is a two-dimensional chain of cadmium, chlorides, and ligands. The ligand uses both of its O atoms and pyridyl N atoms to bond to multiple Cd atoms. In Langley, several manganese clusters (with six, seven, and ten manganese atoms) were studied, all having the enol form of the ligand. The ligands vary their coordination, sometimes bonding in a bidentate fashion *via* the two oxygens, sometimes bidentate with a pyridine nitro­gen and enol oxygen, and sometimes even monodentate *via* the pyridine nitro­gen. Through its multiple modes of bonding in these clusters, the ligand can bond from two to four metal centers.

The ligand has also been shown to use its O atoms and one pyridyl N atom to form a bridging dilanthium complex (Brück *et al.*, 2000 ▸) and a bridging triholmium species (Andrews *et al.*, 2009 ▸). Finally, the ligand has formed a simpler tris­[1,3-bis­(pyridin-2-yl)propane-1,3-dionato]iron(III) compound where the ligand simply bonds to the iron via its O atoms (Lee *et al.*, 2017 ▸). Whereas protonation of pyridyl rings on ligands are common in the literature, this structure is the first to display pyridyl protonation for this particular ligand.

## Synthesis and crystallization   

All chemicals were used as received. To 0.1458 g (0.5 mmol) of nickel bromide hydrate (Aldrich) in 35 ml of water was added 0.2424 g (1.0 mmol) of 1,3-di(2-pyridyl)-1,3-propanedione (TCI) under stirring. To this mixture, concentrated HBr (Fisher) was added dropwise until all the ligand had dissolved (pH ∼ 1). This solution was stirred at room temperature for 30 min and filtered to afford an olive-colored solution. Slow evaporation for 28 d yielded pale-red–orange crystals of the title compound. The yield of the product was 64%. The crystals decomposed when a melting-point determination was attempted. FT–IR data for the free ligand and the title compound are included as supporting information and the appearance of a broad band at 3300 cm^−1^ and a broad band with fine structure at 3000 cm^−1^ confirms the presence of water molecules and pyridinium rings.

## Refinement   

Crystal data, data collection and structure refinement details are summarized in Table 3 ▸. H atoms on *sp* ^2^-hybridized C and N atoms were included in calculated positions, with C—H distances of 0.93 Å and *U*_iso_(H) = 1.2*U*_eq_(C). Water H atoms were refined applying a distance restraint of 0.84 (2) Å.

## Supplementary Material

Crystal structure: contains datablock(s) I. DOI: 10.1107/S205698902000081X/zl2768sup1.cif

Structure factors: contains datablock(s) I. DOI: 10.1107/S205698902000081X/zl2768Isup2.hkl

IR spectrum of NI(dppo). DOI: 10.1107/S205698902000081X/zl2768sup3.pdf

CCDC reference: 1979583

Additional supporting information:  crystallographic information; 3D view; checkCIF report

## Figures and Tables

**Table 1:** Selected geometric parameters (Å, °)

Ni1—O1	2.003 (2)	Ni1—O6	2.080 (3)
Ni1—O2	2.006 (2)	Ni1—O5	2.088 (3)
Ni1—O3	2.031 (2)	Ni1—O4	2.088 (2)
			
O1—Ni1—O2	88.75 (9)	O3—Ni1—O5	93.21 (12)
O1—Ni1—O3	176.19 (9)	O6—Ni1—O5	176.52 (11)
O2—Ni1—O3	87.65 (9)	O1—Ni1—O4	90.82 (9)
O1—Ni1—O6	91.18 (10)	O2—Ni1—O4	177.08 (11)
O2—Ni1—O6	89.88 (10)	O3—Ni1—O4	92.72 (10)
O3—Ni1—O6	87.57 (12)	O6—Ni1—O4	87.24 (11)
O1—Ni1—O5	88.26 (11)	O5—Ni1—O4	89.34 (12)
O2—Ni1—O5	93.54 (11)		

**Table 2:** Hydrogen-bond geometry (Å, °)

*D*—H⋯*A*	*D*—H	H⋯*A*	*D*⋯*A*	*D*—H⋯*A*
N1—H1⋯Br1	0.86	2.94	3.774 (4)	165
N2—H2⋯Br1	0.86	3.02	3.852 (3)	165
C1—H1*A*⋯Br2	0.93	2.75	3.567 (3)	147
C4—H4⋯Br3^i^	0.93	2.47	3.330 (3)	154
C10—H10⋯Br2^ii^	0.93	2.48	3.305 (3)	149
O3—H3*A*⋯Br2^ii^	0.84 (2)	2.48 (4)	3.285 (3)	161 (9)
O3—H3*B*⋯Br3^iii^	0.83 (2)	2.40 (3)	3.212 (2)	165 (8)
O4—H4*A*⋯O7^iv^	0.84 (2)	2.16 (5)	2.917 (4)	150 (9)
O4—H4*B*⋯Br3^i^	0.82 (2)	2.48 (3)	3.284 (3)	167 (9)
O5—H5*A*⋯O7	0.84 (2)	1.99 (3)	2.808 (5)	163 (10)
O5—H5*B*⋯O8	0.84 (2)	2.29 (6)	3.002 (6)	142 (9)
O6—H6*A*⋯Br1^v^	0.84 (2)	2.51 (2)	3.342 (3)	175 (9)
O6—H6*B*⋯Br2^v^	0.83 (2)	2.45 (3)	3.266 (3)	165 (9)
O7—H7*A*⋯Br1^i^	0.84 (2)	2.59 (6)	3.335 (4)	149 (9)
O7—H7*B*⋯Br3^i^	0.85 (2)	2.54 (2)	3.386 (3)	173 (9)
O8—H8*A*⋯Br3^vi^	0.85 (2)	3.06 (3)	3.880 (7)	165 (9)
O8—H8*B*⋯Br1^iii^	0.84 (2)	2.65 (6)	3.393 (5)	149 (9)

Symmetry codes: (i) 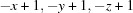; (ii) 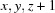; (iii) 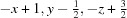; (iv) 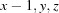; (v) 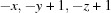; (vi) 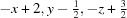.

**Table 3:** Experimental details

Crystal data	
Chemical formula	[Ni(C_13_H_11_N_2_O_2_)(H_2_O)_4_]Br_3_·2H_2_O
*M*_r_	633.77
Crystal system, space group	Monoclinic, *P*2_1_/*c*
Temperature (K)	293
*a*, *b*, *c* (Å)	6.8071 (6), 23.8031 (16), 13.6302 (10)
β (°)	97.476 (9)
*V* (Å^3^)	2189.7 (3)
*Z*	4
Radiation type	Mo *K*α
μ (mm^−1^)	6.40
Crystal size (mm)	0.32 × 0.28 × 0.19

Data collection	
Diffractometer	Agilent Xcalibur Sapphire3
Absorption correction	Multi-scan (*CrysAlis PRO*; Oxford Diffraction, 2009 ▸)
*T*_min_, *T*_max_	0.504, 1.000
No. of measured, independent and observed [*I* > 2σ(*I*)] reflections	26929, 7970, 5803
*R*_int_	0.034
(sin θ/λ)_max_ (Å^−1^)	0.781

Refinement	
*R*[*F* ^2^ > 2σ(*F* ^2^)], *wR*(*F* ^2^), *S*	0.043, 0.125, 1.05
No. of reflections	7970
No. of parameters	280
No. of restraints	12
H-atom treatment	H atoms treated by a mixture of independent and constrained refinement
Δρ_max_, Δρ_min_ (e Å^−3^)	1.10, −1.39

Computer programs: *CrysAlis CCD* and *CrysAlis RED* (Oxford Diffraction, 2009 ▸), *SHELXS2014* (Sheldrick, 2008 ▸), *SHELXL2014* (Sheldrick, 2015 ▸), *ORTEP-3 for Windows* (Farrugia, 2012 ▸), *Mercury* (Macrae *et al.*, 2020 ▸) and *OLEX2* (Bourhis *et al.*, 2015 ▸).

## supplementary crystallographic information

### Crystal data

**Table:**

[Ni(C_13_H_11_N_2_O_2_)(H_2_O)_4_]3Br·2H_2_O	*F*(000) = 1248
*M**_r_* = 633.77	*D*_x_ = 1.922 Mg m^−^^3^
Monoclinic, *P*2_1_/*c*	Mo *K*α radiation, λ = 0.71073 Å
*a* = 6.8071 (6) Å	Cell parameters from 4741 reflections
*b* = 23.8031 (16) Å	θ = 4.6–32.1°
*c* = 13.6302 (10) Å	µ = 6.40 mm^−^^1^
β = 97.476 (9)°	*T* = 293 K
*V* = 2189.7 (3) Å^3^	Block, orange
*Z* = 4	0.32 × 0.28 × 0.19 mm

### Data collection

**Table:**

Agilent Xcalibur Sapphire3 diffractometer	7970 independent reflections
Radiation source: Enhance (Mo) X-ray Source	5803 reflections with *I* > 2σ(*I*)
Detector resolution: 16.1790 pixels mm^-1^	*R*_int_ = 0.034
ω scans	θ_max_ = 33.7°, θ_min_ = 4.3°
Absorption correction: multi-scan (CrysAlis PRO; Oxford Diffraction, 2009)	*h* = −10→10
*T*_min_ = 0.503, *T*_max_ = 1.000	*k* = −36→36
26929 measured reflections	*l* = −20→21

### Refinement

**Table:**

Refinement on *F*^2^	Primary atom site location: structure-invariant direct methods
Least-squares matrix: full	Secondary atom site location: difference Fourier map
*R*[*F*^2^ > 2σ(*F*^2^)] = 0.043	Hydrogen site location: mixed
*wR*(*F*^2^) = 0.125	H atoms treated by a mixture of independent and constrained refinement
*S* = 1.05	*w* = 1/[σ^2^(*F*_o_^2^) + (0.0561*P*)^2^ + 2.3402*P*] where *P* = (*F*_o_^2^ + 2*F*_c_^2^)/3
7970 reflections	(Δ/σ)_max_ = 0.001
280 parameters	Δρ_max_ = 1.10 e Å^−^^3^
12 restraints	Δρ_min_ = −1.39 e Å^−^^3^

### Special details

**Table:**

Geometry. All esds (except the esd in the dihedral angle between two l.s. planes) are estimated using the full covariance matrix. The cell esds are taken into account individually in the estimation of esds in distances, angles and torsion angles; correlations between esds in cell parameters are only used when they are defined by crystal symmetry. An approximate (isotropic) treatment of cell esds is used for estimating esds involving l.s. planes.

### Fractional atomic coordinates and isotropic or equivalent isotropic displacement parameters (Å^2^)

**Table:**

	*x*	*y*	*z*	*U*_iso_*/*U*_eq_	
Br1	0.28363 (5)	0.60904 (2)	0.46388 (3)	0.04291 (10)	
Br2	0.22784 (5)	0.48206 (2)	0.10507 (2)	0.03503 (9)	
Br3	0.80885 (8)	0.76767 (2)	0.39510 (3)	0.05624 (13)	
Ni1	0.15038 (6)	0.37164 (2)	0.81987 (3)	0.02833 (10)	
O1	0.2089 (4)	0.37484 (8)	0.67975 (15)	0.0311 (4)	
O2	0.2542 (4)	0.45031 (9)	0.83901 (15)	0.0318 (5)	
O3	0.0917 (5)	0.37407 (10)	0.96216 (18)	0.0439 (6)	
O4	0.0286 (4)	0.29148 (10)	0.79662 (19)	0.0417 (6)	
O5	0.4287 (5)	0.33525 (12)	0.8585 (2)	0.0489 (6)	
O6	−0.1333 (4)	0.40312 (11)	0.77851 (19)	0.0414 (5)	
N1	0.2313 (5)	0.45128 (15)	0.4508 (2)	0.0488 (8)	
H1	0.2437	0.4862	0.4669	0.059*	
N2	0.3028 (5)	0.59168 (13)	0.7459 (2)	0.0473 (7)	
H2	0.3017	0.5882	0.6831	0.057*	
C1	0.2240 (6)	0.43458 (18)	0.3530 (2)	0.0464 (9)	
H1A	0.2312	0.4615	0.3041	0.056*	
C2	0.2062 (7)	0.37877 (18)	0.3273 (2)	0.0480 (9)	
H2A	0.2029	0.3677	0.2617	0.058*	
C3	0.1935 (6)	0.33996 (17)	0.3999 (2)	0.0432 (8)	
H3	0.1814	0.3020	0.3841	0.052*	
C4	0.1984 (4)	0.35680 (12)	0.49387 (18)	0.0241 (5)	
H4	0.1874	0.3297	0.5420	0.029*	
C5	0.2182 (4)	0.41042 (12)	0.52228 (19)	0.0267 (5)	
C6	0.2239 (5)	0.41925 (12)	0.6312 (2)	0.0267 (5)	
C7	0.2441 (5)	0.47393 (12)	0.6692 (2)	0.0300 (6)	
H7	0.2477	0.5037	0.6254	0.036*	
C8	0.2593 (4)	0.48545 (11)	0.7704 (2)	0.0264 (5)	
C9	0.2832 (4)	0.54534 (11)	0.8042 (2)	0.0246 (5)	
C10	0.2865 (4)	0.55252 (11)	0.90202 (18)	0.0216 (5)	
H10	0.2701	0.5211	0.9408	0.026*	
C11	0.3121 (6)	0.60228 (14)	0.9464 (3)	0.0387 (7)	
H11	0.3170	0.6051	1.0148	0.046*	
C12	0.3313 (6)	0.64942 (15)	0.8900 (3)	0.0469 (9)	
H12	0.3490	0.6845	0.9198	0.056*	
C13	0.3243 (6)	0.64421 (14)	0.7904 (3)	0.0450 (8)	
H13	0.3339	0.6760	0.7517	0.054*	
O7	0.6193 (5)	0.29510 (15)	0.7017 (3)	0.0586 (8)	
O8	0.6929 (11)	0.2404 (2)	0.9388 (4)	0.1057 (17)	
H3A	0.120 (13)	0.4061 (18)	0.986 (6)	0.159*	
H3B	0.140 (12)	0.349 (3)	1.000 (5)	0.159*	
H4A	−0.095 (3)	0.289 (4)	0.791 (7)	0.159*	
H4B	0.061 (14)	0.272 (3)	0.752 (5)	0.159*	
H5A	0.489 (13)	0.317 (4)	0.818 (6)	0.159*	
H5B	0.448 (15)	0.304 (2)	0.886 (7)	0.159*	
H6A	−0.169 (13)	0.402 (4)	0.7173 (18)	0.159*	
H6B	−0.178 (13)	0.432 (2)	0.802 (7)	0.159*	
H7A	0.592 (15)	0.319 (3)	0.656 (5)	0.159*	
H7B	0.513 (8)	0.281 (4)	0.673 (7)	0.159*	
H8A	0.789 (10)	0.252 (4)	0.980 (6)	0.159*	
H8B	0.658 (15)	0.2068 (16)	0.945 (8)	0.159*	

### Atomic displacement parameters (Å^2^)

**Table:**

	*U*^11^	*U*^22^	*U*^33^	*U*^12^	*U*^13^	*U*^23^
Br1	0.03826 (18)	0.0478 (2)	0.04137 (18)	−0.00130 (14)	0.00006 (14)	0.00498 (15)
Br2	0.04109 (18)	0.04094 (18)	0.02300 (13)	0.00484 (13)	0.00390 (12)	−0.00060 (11)
Br3	0.0857 (3)	0.02834 (17)	0.0548 (2)	−0.00371 (17)	0.0097 (2)	−0.01026 (15)
Ni1	0.0437 (2)	0.02121 (17)	0.02022 (16)	−0.00070 (14)	0.00474 (15)	0.00139 (12)
O1	0.0497 (13)	0.0228 (9)	0.0210 (9)	0.0008 (9)	0.0051 (9)	0.0011 (7)
O2	0.0510 (13)	0.0230 (9)	0.0213 (9)	−0.0049 (9)	0.0038 (9)	0.0000 (7)
O3	0.0761 (19)	0.0318 (12)	0.0258 (10)	−0.0031 (12)	0.0139 (12)	0.0026 (9)
O4	0.0597 (16)	0.0247 (10)	0.0418 (13)	−0.0045 (11)	0.0105 (12)	−0.0001 (9)
O5	0.0583 (17)	0.0447 (15)	0.0407 (13)	0.0133 (13)	−0.0053 (12)	0.0019 (11)
O6	0.0482 (14)	0.0358 (12)	0.0392 (12)	0.0026 (11)	0.0021 (11)	−0.0038 (10)
N1	0.064 (2)	0.0480 (18)	0.0351 (15)	0.0050 (16)	0.0085 (15)	0.0064 (13)
N2	0.064 (2)	0.0373 (15)	0.0405 (15)	−0.0065 (15)	0.0061 (15)	−0.0003 (13)
C1	0.061 (2)	0.055 (2)	0.0230 (13)	0.0033 (18)	0.0076 (15)	0.0093 (14)
C2	0.058 (2)	0.066 (3)	0.0200 (13)	0.0027 (19)	0.0057 (15)	−0.0077 (14)
C3	0.054 (2)	0.0456 (19)	0.0301 (15)	−0.0022 (16)	0.0045 (15)	−0.0164 (14)
C4	0.0314 (13)	0.0240 (11)	0.0170 (10)	−0.0010 (10)	0.0033 (10)	−0.0048 (9)
C5	0.0314 (14)	0.0292 (13)	0.0193 (10)	0.0021 (11)	0.0029 (10)	−0.0013 (10)
C6	0.0349 (14)	0.0250 (12)	0.0201 (10)	0.0019 (11)	0.0038 (10)	0.0000 (9)
C7	0.0476 (17)	0.0219 (12)	0.0204 (11)	−0.0025 (11)	0.0040 (12)	0.0018 (9)
C8	0.0341 (14)	0.0219 (11)	0.0227 (11)	−0.0020 (10)	0.0021 (11)	−0.0011 (9)
C9	0.0275 (12)	0.0226 (12)	0.0232 (11)	−0.0021 (10)	0.0022 (10)	−0.0023 (9)
C10	0.0260 (12)	0.0190 (11)	0.0202 (10)	−0.0032 (9)	0.0052 (9)	−0.0035 (8)
C11	0.0496 (19)	0.0339 (16)	0.0346 (15)	−0.0085 (14)	0.0135 (15)	−0.0141 (13)
C12	0.062 (2)	0.0276 (16)	0.053 (2)	−0.0092 (15)	0.0142 (18)	−0.0108 (14)
C13	0.065 (2)	0.0242 (14)	0.0465 (19)	−0.0097 (15)	0.0092 (18)	0.0011 (14)
O7	0.0591 (18)	0.0536 (18)	0.0612 (19)	0.0006 (14)	0.0002 (15)	0.0041 (14)
O8	0.161 (5)	0.071 (3)	0.086 (3)	0.024 (3)	0.020 (3)	0.020 (3)

### Geometric parameters (Å, º)

**Table:**

Ni1—O1	2.003 (2)	C2—C3	1.365 (6)
Ni1—O2	2.006 (2)	C2—H2A	0.9300
Ni1—O3	2.031 (2)	C3—C4	1.339 (4)
Ni1—O6	2.080 (3)	C3—H3	0.9300
Ni1—O5	2.088 (3)	C4—C5	1.336 (4)
Ni1—O4	2.088 (2)	C4—H4	0.9300
O1—C6	1.258 (3)	C5—C6	1.495 (4)
O2—C8	1.259 (3)	C6—C7	1.401 (4)
O3—H3A	0.84 (2)	C7—C8	1.396 (4)
O3—H3B	0.83 (2)	C7—H7	0.9300
O4—H4A	0.84 (2)	C8—C9	1.501 (4)
O4—H4B	0.82 (2)	C9—C10	1.341 (3)
O5—H5A	0.84 (2)	C10—C11	1.331 (4)
O5—H5B	0.84 (2)	C10—H10	0.9300
O6—H6A	0.84 (2)	C11—C12	1.376 (5)
O6—H6B	0.83 (2)	C11—H11	0.9300
N1—C1	1.386 (5)	C12—C13	1.358 (5)
N1—C5	1.387 (4)	C12—H12	0.9300
N1—H1	0.8600	C13—H13	0.9300
N2—C9	1.376 (4)	O7—H7A	0.84 (2)
N2—C13	1.389 (5)	O7—H7B	0.85 (2)
N2—H2	0.8600	O8—H8A	0.85 (2)
C1—C2	1.375 (6)	O8—H8B	0.84 (2)
C1—H1A	0.9300		
			
O1—Ni1—O2	88.75 (9)	C3—C2—C1	118.7 (3)
O1—Ni1—O3	176.19 (9)	C3—C2—H2A	120.6
O2—Ni1—O3	87.65 (9)	C1—C2—H2A	120.6
O1—Ni1—O6	91.18 (10)	C4—C3—C2	119.7 (3)
O2—Ni1—O6	89.88 (10)	C4—C3—H3	120.2
O3—Ni1—O6	87.57 (12)	C2—C3—H3	120.2
O1—Ni1—O5	88.26 (11)	C5—C4—C3	123.5 (3)
O2—Ni1—O5	93.54 (11)	C5—C4—H4	118.2
O3—Ni1—O5	93.21 (12)	C3—C4—H4	118.2
O6—Ni1—O5	176.52 (11)	C4—C5—N1	118.7 (3)
O1—Ni1—O4	90.82 (9)	C4—C5—C6	114.3 (2)
O2—Ni1—O4	177.08 (11)	N1—C5—C6	127.1 (3)
O3—Ni1—O4	92.72 (10)	O1—C6—C7	126.6 (3)
O6—Ni1—O4	87.24 (11)	O1—C6—C5	114.3 (2)
O5—Ni1—O4	89.34 (12)	C7—C6—C5	119.1 (2)
C6—O1—Ni1	124.99 (19)	C8—C7—C6	122.6 (3)
C8—O2—Ni1	124.54 (18)	C8—C7—H7	118.7
Ni1—O3—H3A	109 (6)	C6—C7—H7	118.7
Ni1—O3—H3B	117 (6)	O2—C8—C7	126.7 (3)
H3A—O3—H3B	112 (9)	O2—C8—C9	114.5 (2)
Ni1—O4—H4A	118 (7)	C7—C8—C9	118.8 (2)
Ni1—O4—H4B	120 (7)	C10—C9—N2	118.8 (3)
H4A—O4—H4B	104 (8)	C10—C9—C8	114.5 (2)
Ni1—O5—H5A	123 (7)	N2—C9—C8	126.8 (3)
Ni1—O5—H5B	124 (7)	C11—C10—C9	123.3 (3)
H5A—O5—H5B	77 (8)	C11—C10—H10	118.3
Ni1—O6—H6A	114 (6)	C9—C10—H10	118.3
Ni1—O6—H6B	125 (7)	C10—C11—C12	119.2 (3)
H6A—O6—H6B	109 (8)	C10—C11—H11	120.4
C1—N1—C5	118.5 (3)	C12—C11—H11	120.4
C1—N1—H1	120.8	C13—C12—C11	119.4 (3)
C5—N1—H1	120.8	C13—C12—H12	120.3
C9—N2—C13	118.8 (3)	C11—C12—H12	120.3
C9—N2—H2	120.6	C12—C13—N2	120.4 (3)
C13—N2—H2	120.6	C12—C13—H13	119.8
C2—C1—N1	120.9 (3)	N2—C13—H13	119.8
C2—C1—H1A	119.5	H7A—O7—H7B	80 (8)
N1—C1—H1A	119.5	H8A—O8—H8B	116 (10)

### Hydrogen-bond geometry (Å, º)

**Table:**

*D*—H···*A*	*D*—H	H···*A*	*D*···*A*	*D*—H···*A*
N1—H1···Br1	0.86	2.94	3.774 (4)	165
N2—H2···Br1	0.86	3.02	3.852 (3)	165
C1—H1*A*···Br2	0.93	2.75	3.567 (3)	147
C4—H4···Br3^i^	0.93	2.47	3.330 (3)	154
C10—H10···Br2^ii^	0.93	2.48	3.305 (3)	149
O3—H3*A*···Br2^ii^	0.84 (2)	2.48 (4)	3.285 (3)	161 (9)
O3—H3*B*···Br3^iii^	0.83 (2)	2.40 (3)	3.212 (2)	165 (8)
O4—H4*A*···O7^iv^	0.84 (2)	2.16 (5)	2.917 (4)	150 (9)
O4—H4*B*···Br3^i^	0.82 (2)	2.48 (3)	3.284 (3)	167 (9)
O5—H5*A*···O7	0.84 (2)	1.99 (3)	2.808 (5)	163 (10)
O5—H5*B*···O8	0.84 (2)	2.29 (6)	3.002 (6)	142 (9)
O6—H6*A*···Br1^v^	0.84 (2)	2.51 (2)	3.342 (3)	175 (9)
O6—H6*B*···Br2^v^	0.83 (2)	2.45 (3)	3.266 (3)	165 (9)
O7—H7*A*···Br1^i^	0.84 (2)	2.59 (6)	3.335 (4)	149 (9)
O7—H7*B*···Br3^i^	0.85 (2)	2.54 (2)	3.386 (3)	173 (9)
O8—H8*A*···Br3^vi^	0.85 (2)	3.06 (3)	3.880 (7)	165 (9)
O8—H8*B*···Br1^iii^	0.84 (2)	2.65 (6)	3.393 (5)	149 (9)

Symmetry codes: (i) −*x*+1, −*y*+1, −*z*+1; (ii) *x*, *y*, *z*+1; (iii) −*x*+1, *y*−1/2, −*z*+3/2; (iv) *x*−1, *y*, *z*; (v) −*x*, −*y*+1, −*z*+1; (vi) −*x*+2, *y*−1/2, −*z*+3/2.
