# Supplementary figures and images for: Tetra­aqua­[3-oxo-1,3-bis­(pyridinium-2-yl)propan-1-olato]nickel(II) tribromide dihydrate
Source: Acta Crystallogr E Crystallogr Commun. 2020 Jan 31;76(Pt 2):270–2. doi: 10.1107/S205698902000081X (PMC7001841; doi:10.1107/S205698902000081X)

IR of Ni(dppo)

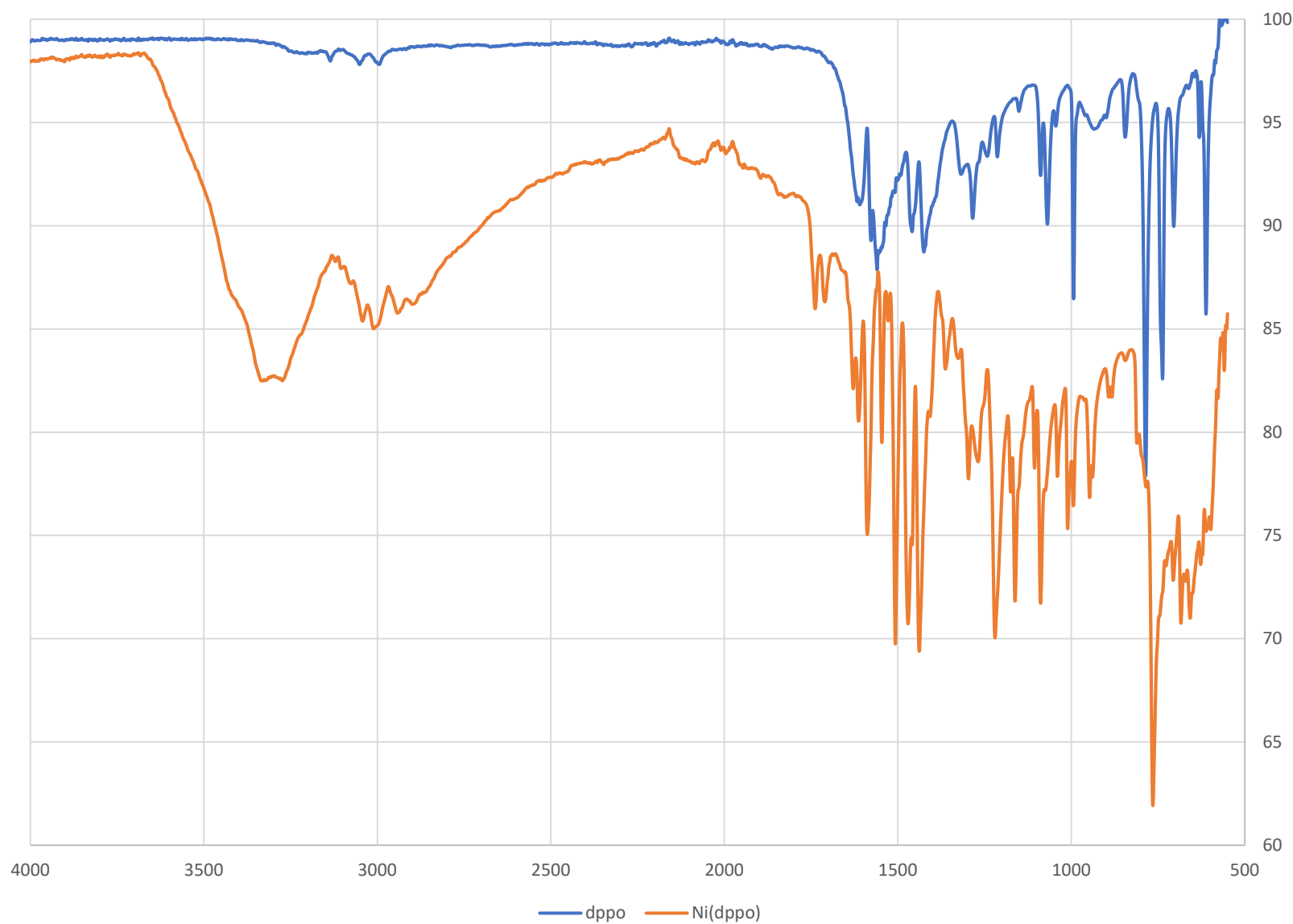

Supplement: Supplementary file 3 [file e-76-00270-sup3.pdf]
